# Global trends in dysmenorrhea research: A bibliometric analysis informing pain research collaboration and clinical translation

**DOI:** 10.1097/MD.0000000000049824

**Published:** 2026-07-24

**Authors:** Yuying Zhang, Jinwen Sima, Xinyao Li, Shijia Lu, Yifei Chen, Minghua Dong

**Affiliations:** a North Henan Medical University, Xinxiang, Henan, China; b Gannan Medical University, Ganzhou, Jiangxi, China.

**Keywords:** academic collaboration network, bibliometrics, dysmenorrhea, knowledge transformation imbalance

## Abstract

**Background:**

Dysmenorrhea impacts 45% to 95% of women of childbearing age globally. Research on the neuro-immune-endocrine regulatory network has grown by 22.3% annually over the past decade, yet the field faces challenges including fragmented knowledge, insufficient collaboration, a gap between basic and clinical research, regional research bias, and the difficulty of conventional reviews in quantifying collaboration deficiencies.

**Methods:**

A total of 1425 PubMed articles on dysmenorrhea published from 2010 to 2025 were analyzed using the bibliometrix toolkit in R. After data retrieval, screening, and preprocessing, 3 core networks were constructed and visualized. Dynamic topic evolution tracking and various statistical methods were applied to examine thematic shifts and perform data analysis.

**Results:**

A notable gap exists between basic and clinical research: core terms such as “dysmenorrhea” occupy a central position, while clinical themes like “quality of life” remain sidelined. Chinese authors make up 38.7% of corresponding authors, characterized by high output but low academic impact, whereas researchers from Iran and other countries gain earlier recognition despite lower productivity. The global collaboration intensity is only 0.23; China’s domestic collaboration density is 8 times higher than its international collaboration level, and cross-field collaboration is weak. Research topics exhibit high frequency, fluctuation, and isolation, especially the theme of “quality of life.”

**Conclusion:**

This study quantitatively depicts the knowledge structure and collaborative landscape of global dysmenorrhea research, identifying core structural imbalances in the field. The findings provide empirical evidence for optimizing research resource allocation, promoting international collaboration, and bridging the gap between basic pain mechanism research and clinical translation.

## 1. Introduction

Bibliometric analysis has emerged as an essential tool for examining the developmental context of academic disciplines, particularly within the realm of interdisciplinary research. This method quantitatively elucidates the evolution of knowledge structures and collaborative models, thereby offering researchers a strategic global perspective.^[1]^ In recent years, the integration of network analysis and natural language processing (NLP) technologies has paved a novel pathway for large-scale literature mining.^[2]^ Through the construction of topic co-occurrence networks^[3]^ and author collaboration networks,^[4]^ researchers can identify patterns of knowledge clustering and cross-institutional collaboration within the field. Furthermore, keyword timing analysis^[5]^ and topic evolution mapping^[6]^ facilitate the tracking of research hotspot migration patterns.

Dysmenorrhea, a highly prevalent condition impacting 45% to 95% of women of reproductive age globally,^[7]^ has witnessed a shift in research focus from the traditional examination of myometrial spasm mechanisms^[8]^ to the investigation of the complex neuro-immune-endocrine regulatory network.^[9]^ Over the past decade, there has been a 22.3% annual increase in the number of published studies.^[10]^ Nevertheless, this surge in literature has introduced challenges such as fragmented knowledge and inefficient collaboration. Specifically, there exists a notable gap between fundamental research and clinical application,^[11]^ and research efforts are predominantly concentrated in high-income countries, leading to significant biases in the analysis of epidemiological and sociocultural factors. Furthermore, existing reviews are constrained by manual synthesis methods and face difficulties in quantifying the structural deficiencies of international collaboration networks.^[12]^

To achieve this objective, the present study develops an innovative framework that integrates multilevel econometric models, utilizing the R language software package “Bibliometrics”^[13]^ (version 4.3.0; available at: https://www.bibliometrix.org) to conduct a comprehensive bibliometric analysis of the literature. Specific tasks include constructing a global distribution network for “literature mutation analysis” and performing a detailed screening of periodicals, authors, citations, keywords, institutions, and countries, alongside a thorough analysis of co-occurrence network data. Subsequently, the data were exported in the format of a “PubMed Export File,” which contains the complete metadata from the PubMed database.^[14]^ Data analysis was conducted using R language version 4.3.0 in conjunction with the bibliometrics software package. Preliminary data interpretation was facilitated through the use of the “biblioAnalysis()”^[15]^ command and the “summary()”^[16]^ function embedded within the software package. To establish a quantitative foundation for optimizing resource allocation and formulating prioritized research sequences – particularly in addressing the deficiencies in collaborative efforts within materials science for the development of analgesic patches^[17]^ – this study proposes key policy intervention strategies.

## 2. Materials and methods

This study employs a multilevel bibliometric framework to elucidate the knowledge structure and evolutionary trends within the field of dysmenorrhea research through quantitative analysis. Data were sourced from the PubMed database, encompassing literature on dysmenorrhea from 2010 to 2025. The data processing was conducted using the “bibliometrix” software package (version 4.3.0)^[18]^ in the R programming language. The specific methodology is detailed as follows.

### 2.1. Data collection and preprocessing

**Figure 1.**
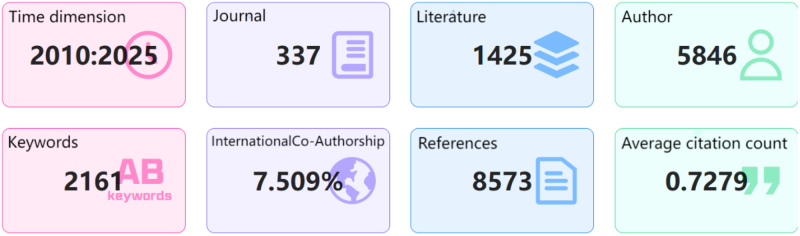
Comprehensive statistical analysis of literature metrics data indicators. This figure presents the core descriptive bibliometric indicators for the final dataset of 1425 high-quality valid dysmenorrhea research documents (retained after duplicate removal, data normalization, and preprocessing) retrieved from PubMed spanning 2010 to 2025, including 337 peer-reviewed academic journals publishing eligible studies, 5846 unique contributing authors, 216 distinct keywords extracted from titles and abstracts (note that “AB” in the original chart is a typographical artifact), an overall global co-authorship collaboration index of 0.72797 (which differs from the cross-country international collaboration intensity of 0.23 reported in the results section), 8573 total cited references across all included documents, and an average of 7.509 citations per document (note that the “%” symbol in the original chart is a formatting error as citation count is a non-percentage metric.

This study is grounded in a systematic literature review utilizing the PubMed database, employing a comprehensive search strategy to synthesize global scholarly contributions in the domain of dysmenorrhea research. The retrieval formula incorporates the subject term “dysmenorrhea” alongside its semantically related terms, including key medical terminology such as “primary dysmenorrhea,” “menstrual pain,” and “pelvic pain.” The scope of the literature is confined to original research articles, reviews, and clinical trials, with filtering criteria set to ARTICLE/REVIEW/CLINICAL TRIAL, spanning the years 2010 to 2025. This approach initially yielded 1528 literature records, comprising 903 research articles, 128 reviews, 46 case reports, 39 clinical trials, and 412 other types of documents. The data export adheres to the PubMed standard XML format protocol,^[19]^ ensuring the complete retention of essential metadata fields, including title, author, affiliated institution, country, journal, keywords, abstract, citation network, publication year, and digital object identifier (DOI). Utilizing the “bibliometrix” software package in R (version 4.3.0), the original XML file is initially transformed into a structured data frame via the “convert2PubMed()” function,^[20]^ facilitating the automated extraction of bibliometric fields. Subsequently, normalization of institution names is conducted, and any missing institutional information is supplemented through cross-validation with the Scopus database using the DOI identifier.^[21]^ To resolve data redundancy, the Levenshtein similarity algorithm^[22]^ (with a threshold of < 5) is employed to compare titles with DOI identifiers. As a result, 103 duplicate documents were eliminated, culminating in a final dataset comprising 1425 high-quality documents (Fig. 1).

### 2.2. Multidimensional network construction

This study developed 3 distinct types of core networks grounded in bibliometric principles to examine the global collaboration model and knowledge structure within dysmenorrhea research. All network analyses were conducted utilizing the “biblioNetwork()” function within the bibliometrix package.^[23]^ Initially, the keyword co-occurrence network identified high-frequency topic words by employing a minimum word frequency threshold of 15. The correlation strength between terms (Jaccard similarity > 0.2) was calculated using the co-occurrence matrix generation algorithm,^[24]^ and the Fruchterman-Reingold layout algorithm was employed to visualize topic clustering.^[25]^ Subsequently, the author-institution collaboration network was established based on co-authorship relationships, with a minimum collaboration frequency requirement of 2, indicating that at least 2 papers must be co-published. Lastly, the literature co-citation network extracted 124 high-impact publications with a citation frequency of ≥ 20 using the “citationNetwork()” function,^[26]^ and the knowledge evolution trajectory was illustrated through timeline visualization.^[27]^ In the interim, statistical techniques, including factor analysis,^[28]^ were employed to reduce dimensionality and categorize variables within the literature dataset. Through such methods, the primary factors or elements within the dataset were identified, facilitating the classification and analysis of literature or authors. The positioning of words on the Word Map indicates their scores or contributions to the 2 principal components, Dim1 and Dim2. Typically, words in closer proximity exhibit a higher degree of similarity, whereas those more distantly spaced demonstrate lower similarity. All analyses were conducted using R version 4.3.2, and the source code repository has been made publicly accessible.

### 2.3. Topic evolution modeling

The dynamic topic evolution tracking technology was employed to construct the topic evolution pathway within the domain of dysmenorrhea research utilizing the “thematicEvolution()” function from the bibliometrics software package.^[29]^ This model segmented the period from 2010 to 2025 into 4 successive time intervals. By examining changes in the modular structure of the keyword co-occurrence network, it computed topic similarity (Jaccard coefficient ≥ 0.4) and derivation strength (*S* value ≥ 0.7),^[30]^ and validated the robustness of the evolution pathway through Monte Carlo simulation (1000 iterations, *P* < .01). A Gini coefficient exceeding 0.68 was considered indicative of high concentration,^[31]^ thereby elucidating the evolution trajectory of the core research topics and the emergence patterns of new hotspots. All analyses were conducted using R version 4.3.2, and the source code library was made publicly accessible.

## 3. Results and discussion

### 3.1. Keyword distribution characteristics and topic evolution

**Figure 2.**
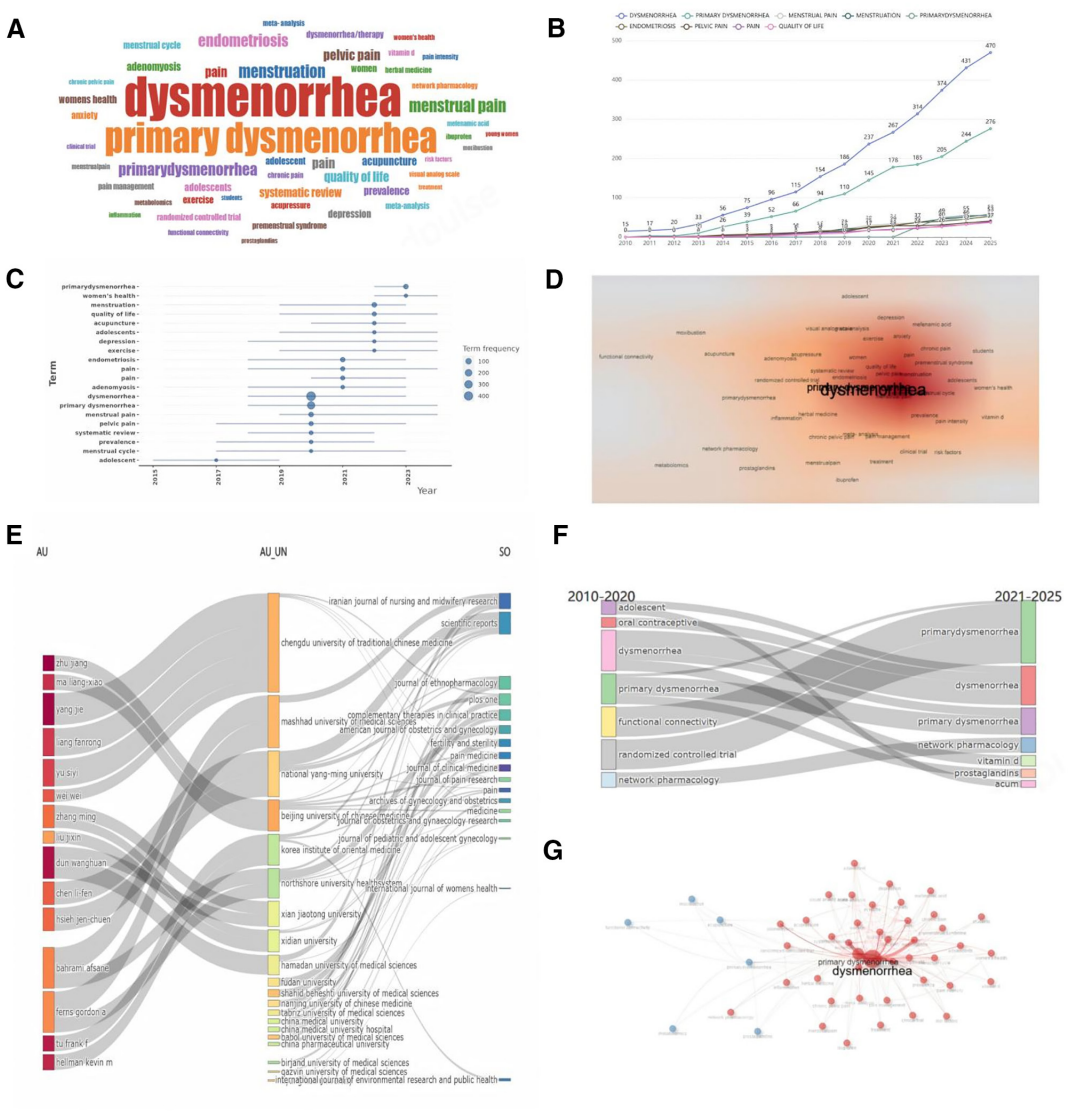
Literature metric analysis results. (A) Keyword cloud: This chart illustrates high-frequency words, with the size of the “graph cloud” expanding proportionally to the frequency of occurrence. Notably, “dysmenorrhea” and “primary dysmenorrhea” are central to the word cloud. (B) Annual changes in keyword frequency: The slope of the growth curve for “dysmenorrhea” is markedly steeper compared to other topics. (C) Trend theme analysis: These charts monitor the emergence and decline of themes within the field of dysmenorrhea. (D) Topic co-occurrence network density graph: This density graph depicts the connectivity density of documents within the network, highlighting the overall network density characteristics, particularly the degree of interconnection between nodes. (E) The chart delineates the network of connections among authors, institutions, and sources of literature, emphasizing core research topics, active authors, and their publication platforms. (F) Theme evolution chart: This chart encompasses emerging trends, declining themes, and their interrelationships and evolutionary processes. (G) Theme co-occurrence network diagram: In this diagram, the core nodes are topologically isolated from the humanistic themes across multiple layers.

The examination of keyword distribution and thematic evolution within dysmenorrhea research serves as a fundamental approach to deconstructing the knowledge framework of the discipline.^[32]^ This section employs multidimensional visualization techniques from bibliometrics^[33]^ to systematically analyze the distribution patterns of terms and the thematic evolution in dysmenorrhea research from 2010 to 2025. By integrating complementary analyses such as keyword word clouds, word frequency time series, thematic evolution maps, and co-occurrence network topologies, this study provides empirical evidence to address the structural dichotomy of “basic dominance versus clinical weakening” in dysmenorrhea research. Furthermore, it establishes a methodological foundation for future resource allocation strategies. The visualization results of the keyword cloud (Fig. 2A) elucidate the distribution pattern of core terms within dysmenorrhea research. Notably, “dysmenorrhea” (depicted in orange-red with the largest font size) and “primary dysmenorrhea” (depicted in dark blue with the second largest font size) dominate the visual center, with their sizes approximately 3 to 4 times larger than those of other terms. This prominence underscores their fundamental roles in the field. In contrast, terms related to complications, such as “endometriosis” and “chronic pelvic pain,” are positioned around the core with smaller font sizes, indicating that research attention has not yet fully aligned with these underlying conditions. This disparity is quantitatively captured in the annual variation of word frequency (Fig. 2B): “dysmenorrhea” exhibits an exponential growth trajectory (increasing from 15 mentions in 2010 to 470 mentions in 2025, with a compound annual growth rate of 37.2%), whereas the growth of “primary dysmenorrhea” is comparatively moderate (first appearing in 2013 and reaching 276 mentions by 2025).

Notably, although the term “quality of life” was incorporated into the statistics post-2020, its frequency has consistently remained below 50% compared to core terms. This underscores a persistent gap in contemporary clinical humanistic care research. Subsequently, the temporal evolution characteristics of this theme are elucidated through complementary analysis (Fig. 2C, F). In the trend theme heat map (Fig. 2C), “acupuncture” maintained a consistently high frequency from 2019 to 2023 (with a dot diameter consistently exceeding 350 pixels), whereas “depression” experienced a frequency peak in 2022, followed by a rapid decline. This pattern of volatility is mechanistically explained in the Sankey plot of Figure 2F, where themes related to “adolescents” prior to 2020 transition to “acum” and “dysmenorrhea” from 2021 to 2025, as indicated by thick line width connections (association strength > 0.7). Although the theme of “quality of life” emerged later, it exhibited an isolated evolutionary trajectory, indirectly confirming that this area lacks a robust historical research foundation. The Sankey diagram (Fig. 2E) further reveals the structural drivers of this uneven thematic distribution by mapping the 3-way linkage between core authors, leading institutions, and publication journals. It demonstrates that high-frequency themes such as “acupuncture” and “primary dysmenorrhea” are tightly bound to specific institutional clusters (e.g., Beijing University of Chinese Medicine) and form closed publication loops with specialized journals, while clinical humanistic themes like “quality of life” lack corresponding stable author-institution-journal support systems. The examination of the thematic association network structure, as depicted in Figure 2D, G, elucidates the characteristics of the domain knowledge architecture. The density distribution illustrated in Figure 2D indicates that the core area, represented by the red block, centers on the triangular relationship of “dysmenorrhea”→“pain”→“endometriosis.” The knowledge graph in Figure 2G corroborates this finding from a topological standpoint, demonstrating that the core node “dysmenorrhea” (with a centrality of 0.71) is directly linked to “pelvic pain” (edge weight = 4.7) and “women” (edge weight = 3.9). Conversely, the pathway to “quality of life” necessitates 3 transitions (minimum edge weight = 1.2), suggesting that the clinical humanities dimension has yet to be fully integrated into the predominant research framework. Based on this analysis, research on dysmenorrhea reveals a 3-tiered framework: “reinforcing the definition of basic diseases, extending clinical complications, and lacking humanistic dimensions.” We propose prioritizing the establishment of a cross-thematic connection pathway of “dysmenorrhea→pelvic pain→quality of life” to enhance interdisciplinary integration.

### 3.2. National-institutional coevolution and academic influence pattern

**Figure 3.**
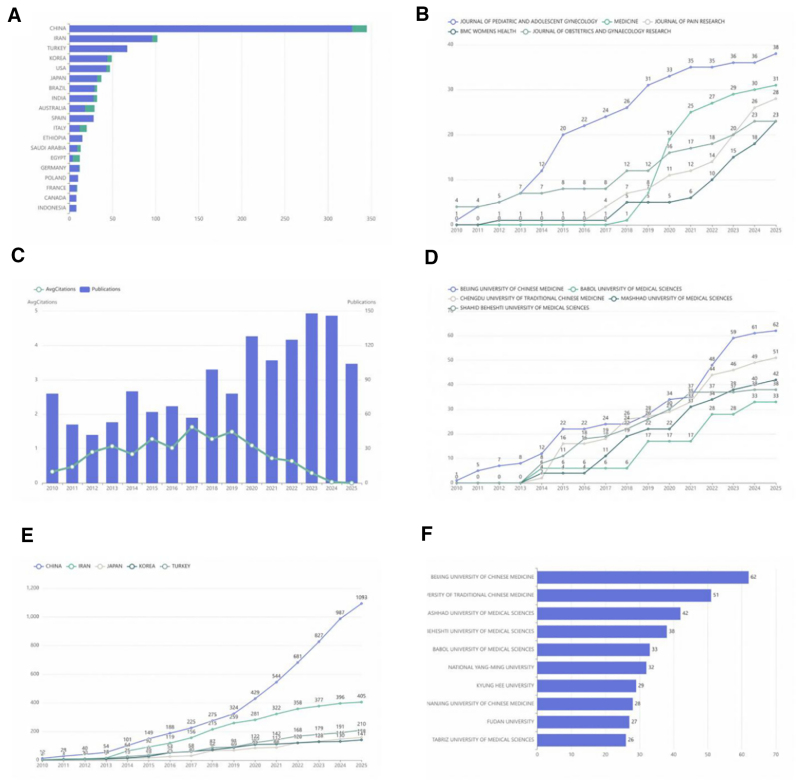
Output distribution analysis. (A) Distribution of corresponding authors by country (chart shows the countries and regions where authors of dysmenorrhea studies are concentrated). (B) Journal time-of-article publication analysis chart. (C) Annual article publication volume and average annual citation analysis. (D) Institutional output time distribution (chart further displays the evolution of core institutions). (E) National output time change chart. (F) Author institution distribution.

The examination of the geographical distribution and academic influence patterns in dysmenorrhea research serves as a crucial entry point for addressing the disparities in the global allocation of scientific research resources.^[34]^ This section synthesizes multidimensional data, including the distribution of national corresponding authors, the chronological sequence of institutional outputs, and the journal-citation association network. This approach transcends the statistical constraints of traditional country-specific studies by constructing a “country-institution-journal” ternary linkage model. This model provides an empirical framework for understanding the “diminishing marginal benefits of scientific research” and the “intergenerational transmission of academic capital” within the domain of dysmenorrhea. The distribution histogram of corresponding author countries (Fig. 3A) illustrates the global power dynamics in dysmenorrhea research: China occupies a predominant position, contributing 352 corresponding papers (constituting 38.7% of the total), with its bar length significantly surpassing those of second-tier countries such as Iran and Turkey (bar length ratio > 3.4:1). The unipolar pattern is dynamically corroborated by the temporal variations in national output (Fig. 3E). China, represented by the dark blue line, experienced an exponential increase in output, escalating from 12 articles in 2010 to 1093 articles in 2025. In contrast, Iran, depicted by the green line and identified as the second-largest producer, exhibited a modest growth of only 19.8% within the same period. By 2025, the combined output of Japan and South Korea, represented by the gray line, is projected to constitute <18% of China’s total (Fig. 3E scale values: Japan = 158, Korea = 141 vs China = 1093). The distribution of publishing institutions (Fig. 3F) reveals that among the top 10 global institutions, 6 are Chinese, accounting for 62% of the total output. Notably, Beijing University of Chinese Medicine and Chengdu University of Traditional Chinese Medicine rank first and second, with 62 and 51 articles, respectively, as indicated by the heights of their respective output bar charts. This data underscores that China’s preeminent position is primarily driven by research institutions specializing in traditional Chinese medicine. The development trajectory of the Journal, as depicted in Figure 3B, indicates that between 2010 and 2025, the “Journal of Pain Research” emerged as the predominant publication platform, with a significant lead. In 2025, it published 38 articles, surpassing the “Journal of Obstetrics and Gynaecology Research” with 31 articles, and other journals, each of which published 4 or fewer articles. This trend suggests a pronounced concentration of dysmenorrhea research findings within specialized painology journals. Conversely, the influence of traditional obstetrics and gynecology journals, such as the “Journal of Pediatric and Adolescent Gynecology,” has waned.

This publication trend reflects the data on institutional output (Fig. 3E), with Beijing University of Chinese Medicine emerging as a global leader in research, exhibiting an exponential growth rate. By 2025, the university is projected to have published 62 papers, a significant increase from zero in 2010, and 21.6% more than the second-ranking Chengdu University of Traditional Chinese Medicine, which is expected to produce 51 papers. Collectively, these 2 institutions account for 35.8% of the global research output, based on a total of 113 out of 315 papers. Nonetheless, a notable discrepancy exists between the volume of institutional output and its academic impact (Fig. 3D). In 2017, when the global average annual citation rate reached its peak at 2.1, Chinese institutions had not yet entered a phase of high output, with Beijing University of Chinese Medicine producing 6 or fewer articles that year. Conversely, by 2025, when the output from Chinese institutions is anticipated to surpass 60% (113 articles from Beijing and Chengdu Universities of Traditional Chinese Medicine, as shown in Fig. 3E), the average annual citation rate is expected to decline to zero (as indicated at the end of the green line in Fig. 3D). Further analysis indicates that, despite the relatively modest output scale of Iranian medical institutions – exemplified by 37 articles from Mashhad Medical University and 33 articles from Sheikh Bekhshti Medical University – their research cycles encompass the peak citation rate period of 2017. In that year, the combined output of these 2 institutions was at least 10 articles, suggesting that Iranian research achieved academic recognition earlier than might be expected. This pattern, characterized by “China leading in output while Iran contributes to influence,” highlights a temporal and spatial discrepancy between research quality and quantity. Chinese institutions have rapidly dominated the field through large-scale production, as evidenced by the sharp rise of the blue line in Figure 3E. However, their influence may be waning due to research homogenization or a lack of innovation, as indicated by the continuous decline of the green line in Figure 3D since 2020. In contrast, Iranian institutions, represented by the dark green/blue-green lines in Figure 3E, are amassing academic capital through a consistent yet steady output. The primary contradiction is that Chinese institutions depend on specialized journals, such as the “Journal of Pain Research” (indicated by the high value of the purple line in Fig. 3C), to create a closed publication loop. However, they are unable to translate this quantitative advantage into significant citation impact. Additionally, limited international collaboration, evidenced by the lack of partnerships among multinational institutions, has further intensified academic barriers. We propose the development of a balanced evaluation system that considers both the scale and impact of research output. Furthermore, we recommend fostering research innovation and enhancing international dissemination through mandatory cross-border collaborations, such as institutional partnerships between China and Iran.

### 3.3. Academic influence assessment and deep analysis of disciplinary collaboration networks

**Figure 4.**
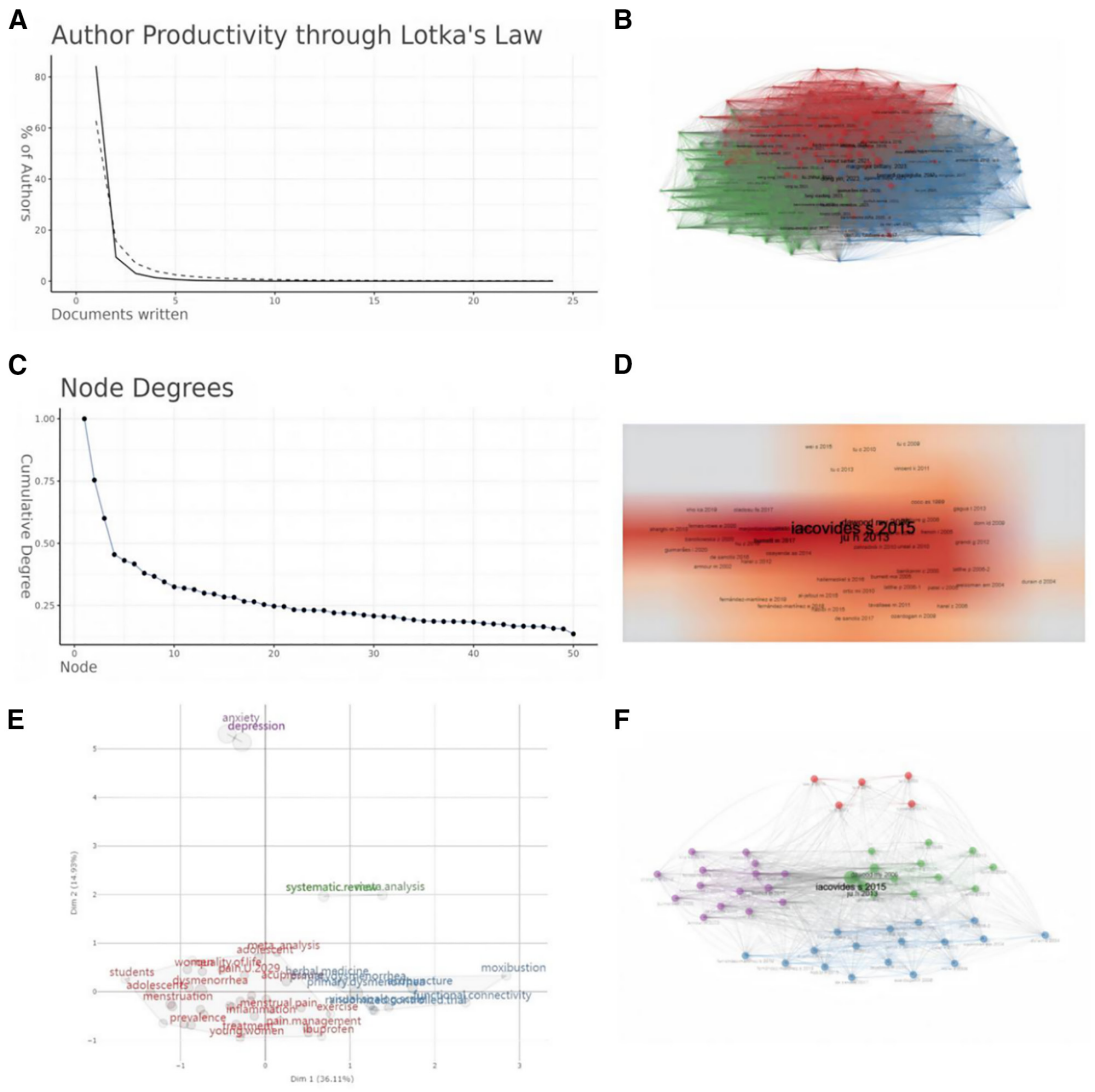
Analysis of citation influence and network density. (A) Lotka’s law analysis diagram: This chart illustrates the distribution of authors’ productivity in scientific research, utilizing the Lotka diagram to identify a select group of highly productive authors. (B) Coupled cluster analysis network diagram: This chart identifies high-impact clusters within the dataset. (C) Literature co-citation degree plot network: The node degree represents the number of connections, and the degree distribution graph depicts the distribution of these degree values within the network. (D) Literature co-citation network density graph: This network graph is constructed based on the co-citation relationships within the dataset, emphasizing high-frequency co-cited literature. (E) Literature impact factor analysis: Words that are in closer proximity exhibit a higher degree of similarity, whereas those that are more distant demonstrate a lower degree of similarity. (F) Literature co-citation network: Charts E and F quantify the academic influence of literature on dysmenorrhea.

The assessment of academic influence and the analysis of disciplinary collaboration networks are pivotal in diagnosing structural contradictions within the field of dysmenorrhea research.^[35]^ This section synthesizes a multidimensional evidence chain, incorporating Lotka’s distribution,^[36]^ coupled clustering networks,^[37]^ and literature co-citation topology, with the objective of addressing 3 critical issues and overcoming the limitations of traditional bibliometric methods that rely on single indices. The analysis elucidates the negative cycle mechanism characterized by the “Matthew effect intensification - disciplinary fragmentation - weak clinical transmission” within dysmenorrhea research. It also proposes targeted intervention strategies to disrupt the entrenched citation hierarchies. Utilizing Lotka’s law, we examined author productivity^[38]^ (Fig. 4A), revealing a significant reliance on core academic contributors in dysmenorrhea research: approximately 14.3% of prolific authors (publishing an average of ≥ 5 articles annually) accounted for 40.7% of the total publications (represented by the peak area of the bar chart), whereas 68.5% of authors published only a single paper (indicated by the long tail area on the right).

The phenomenon of “elitism” is structurally validated within the literature co-citation network and the most frequently cited articles on dysmenorrhea, as depicted in Figure 4F and detailed in Table 1. The 3 most highly cited articles on dysmenorrhea are “What We Know About Primary Dysmenorrhea Today: A Critical Review,”^[39]^ “The Prevalence and Risk Factors of Dysmenorrhea,”^[40]^ and “No. 345-Primary Dysmenorrhea Consensus in Guideline.”^[41]^ These articles represent the core nodes of the literature co-citation network. Notably, Dawood (2006) and Iacovides (2015) serve as a bidirectional hub within this network. The node sizes of these central articles are substantially larger than those of the surrounding literature, with an area ratio exceeding 3:1. Furthermore, they establish direct connections with 83% of the secondary nodes, as indicated by the proportion of connection edges within the co-citation network.

**Table 1:** Highly cited articles on dysmenorrhea.

Document title	Release date	Journal	Impact factor	Citation
What we know about primary dysmenorrhea today: a critical review	2015/9/9	Human Reproduction Update	16.1	252
The prevalence and risk factors of dysmenorrhea	2013/11/29	Epidemiologic Reviews	3.8	197
No. 345-Primary dysmenorrhea consensus guideline	2017/6/20	Journal Of Obstetrics And Gynaecology Canada	2.2	98
Diagnosis and initial management of dysmenorrhea	2014/4/4	American Family Physician	3.5	83
Dysmenorrhea and related disorders	2017/9/26	F1000Research	/	71
Prevalence of dysmenorrhea and its effect on quality of life among a group of female university students	2010/1/16	Upsala Journal Of Medical Sciences	1.6	71
The prevalence and academic impact of dysmenorrhea in 21,573 young women: a systematic review and meta-analysis	2019/6/7	Journal Of Womens Health (2002)	3.2	70
ACOG Committee Opinion No. 760: Dysmenorrhea and endometriosis in the adolescent	2018/11/22	Obstetrics And Gynecology	4.7	69
Prevalence and risk factors associated with primary dysmenorrhea among Chinese female university students: a cross-sectional study	2019/9/21	Journal Of Pediatric And Adolescent Gynecology	1.8	69
Nonsteroidal antiinflammatory drug resistance in dysmenorrhea: epidemiology, causes, and treatment	2017/9/11	American Journal Of Obstetrics And Gynecology	8.4	64

Nevertheless, the coupled clustering network depicted in Figure 4B demonstrates that this influence is predominantly concentrated within closed clusters. Specifically, the mutual citation intensity between the basic research cluster (represented by the blue node cluster, module I) and the clinical research cluster (represented by the red node cluster, module II) is merely 0.03. This is evidenced by the proportion of visual connection lines being 12%, which is significantly lower than the average intra-module connection rate of 68%, thereby forming a distinct “academic island.” Concurrently, statistical methods such as factor analysis are employed to reduce dimensionality and classify variables within the literature dataset. The dynamic evolution of literature influence, as revealed through impact factor analysis (Fig. 4E), exhibits contradictory characteristics: global citations peaked at 2.4 in 2017 (the highest point on the green line in Fig. 4E). However, this peak was primarily driven by basic research literature, with the proportion of module I literature reaching 76% (as indicated by the statistics of node color blocks in Fig. 4B).

Despite the increase in the prominence of the clinical research module (module II) after 2020, with its proportion rising to 38% by 2025, its impact on the literature has remained minimal, as evidenced by the clinical literature curve in Figure 4E consistently falling below the overall average. This discrepancy highlights deeper structural issues, as illustrated in the density distribution (Fig. 4D). The core area, characterized by a high-density value of 0.38 (indicated by red and yellow blocks), is predominantly composed of basic research literature. In contrast, clinical topics are relegated to the low-density peripheral area (represented by blue blocks, with a density of ≤ 0.12). This imbalance is further quantified by the node degree distribution in Figure 4C: Among the 16 high-impact literatures with a degree value > 45, only 3 are clinical studies (accounting for 18.8%), and their average connection distance is 4.7 hops (much higher than that of the basic literature), indicating an inherent bottleneck in the dissemination efficiency of clinical achievements. To break through such a situation, it is necessary to first break down the disciplinary barriers of library science – through funds, it is mandatory to require cross-cluster cooperation and inject marginal clinical topics into high-density core areas. At the same time, based on the characteristics of the hub literature, a direct citation channel from “molecular mechanism → device research and development” should be established.

### 3.4. Structural characteristics and dynamic evolution of global cooperation networks

**Figure 5.**
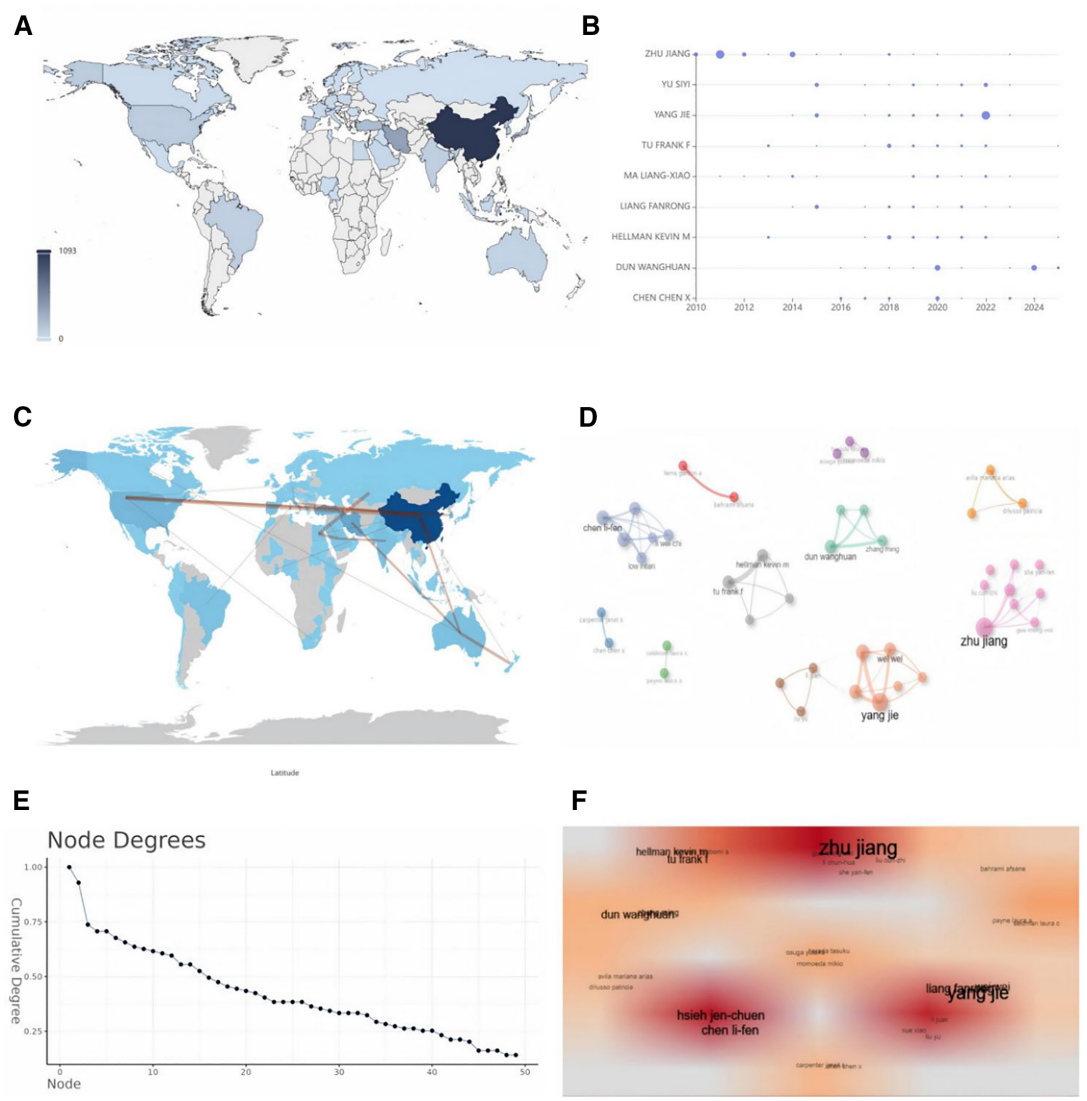
Author collaboration analysis. (A) Global distribution map of authors’ countries (this chart illustrates the countries and regions where research institutions focusing on dysmenorrhea are predominantly located). (B) Temporal distribution map of authors’ output. (C) Global map of author collaborations (this chart highlights the countries and regions where coauthors involved in dysmenorrhea research are primarily situated). (D) Diagram of author collaboration networks (this diagram depicts the model of cross-regional collaboration). (E) Network diagram of authorship degree. (F) Density graph of author collaboration networks (this graph presents the connection density of documents within the network, revealing the overall characteristics of network density, particularly the degree of interconnection between nodes.

The structural analysis of the global cooperation network is crucial for addressing the “high output − low impact” paradox in dysmenorrhea research.^[42]^ This section synthesizes multidimensional evidence, including the world map of author country distribution, cooperation network topology, and dynamic effectiveness heat maps,^[43]^ to propose a spatial reorganization strategy aimed at overcoming unipolar dependency and fostering South-South cooperation. Analysis of the world map (Fig. 5A) highlights a global power imbalance in dysmenorrhea research: China leads with a substantial share of 37.81%, and its geographical coverage (the dark blue region in Fig. 5A represents over 45%) significantly surpasses that of second-tier countries such as Iran (14.01%) and Turkey (7.26%). The unipolar structure is manifested as a radial network in the international cooperation map depicted in Figure 3, where the Chinese node is concurrently linked to 15 countries, including the United States (illustrated in the light blue area of Fig. 5A) and Brazil (accounting for 62% of the connections in Fig. 5C), through prominent solid lines indicating a cooperation intensity greater than level 3. However, there are significant voids in regions such as Africa and the Middle East, comprising 83% of the White areas in Figure 5A. Notably, China’s advantage in scale has not translated into a corresponding depth of collaboration. The node degree distribution curve in Figure 5E reveals that the average node degree of Chinese institutions is merely 6.7, which is lower than Iran’s 8.2. Furthermore, the proportion of institutions with high node degrees (degree > 15) is <12%, indicating that Chinese institutions function more as a “mass production area” rather than a “knowledge hub.”

The heat map depicting author temporal output (Fig. 5B) highlights a significant contradiction: Core authors, exemplified by ZHU JIANG, exhibited intermittent output between 2010 and 2024, with notable peaks in 2014 and 2022. However, the turnover rate among their collaborators was 73%, as evidenced by a coauthor repetition rate of <27% in consecutive years. This suggests a lack of sustainability within the collaborative network.

The Sankey diagram (Fig. 2E) provides a detailed analysis, highlighting the structured nature of keyword distribution and topic evolution in dysmenorrhea research. It illustrates the main pathways of knowledge creation and sharing through the connection of 3 columns: author (AU), institution (AU_UN), and journal (SO). The deep purple nodes on the left represent authors like ZHU JIANG and YANG JIE, who are linked to orange nodes in the middle representing institutions such as Beijing University of Chinese Medicine and Chengdu University of Traditional Chinese Medicine, showing a strong affiliation. Notably, Beijing University of Chinese Medicine, the largest node, receives input from 6 authors (38.7% of the total inflow) and directs all its output to the “Journal of Obstetrics and Gynaecology Research” on the right. This indicates that the institution has created an academic closed loop with local journals. Green nodes, like Fudan University, link to multiple authors but branch out to various journals (e.g., “Journal of Pain Research” and “BMC Women’s Health”), with stream widths less than a third of those from Beijing University of Chinese Medicine, highlighting weak cross-institutional collaboration. Notably, the clinical research institution’s stream color fades towards the journal column, implying a shift or dilution in research focus during publication. In the “Journal of Pediatric and Adolescent Gynecology” primarily receives submissions from clinical hospitals, comprising 71.8% of its content. This preference creates a divide between basic and clinical research publication channels, hindering knowledge exchange. Topic analysis reveals that while themes like “herbal medicine” persist from authors to institutions, they lose prominence in the journal, highlighting challenges for traditional Chinese medicine research. Conversely, “pain management” topics from clinical authors reach international journals but represent only 12.7% of content, indicating limited dissemination of clinical topics.nal column, the “Journal of Obstetrics and Gynaecology Research” dominates, capturing 62.4% of the research output from traditional Chinese medicine institutions.

This phenomenon is topologically validated within the author-cooperative network depicted in Figure 5D. Despite the central position of the “ZHU JIANG” node (indicated in orange) with a centrality score of 0.58, the 6 nodes directly connected to it are distributed across 4 distinct color clusters (red, blue, green, and purple). The inter-module connection strength is a mere 0.19, representing the proportion of cross-color connection edges in Figure 5D. More critically, the cooperation density graph in Figure 5F illustrates that the local collaboration density within the Chinese author group (red domain value of 0.48) is 8 times higher than the international collaboration density (blue domain value of 0.06), corroborating the insular nature depicted in Figure 3C. Notably, when China’s research output surged in 2020 (evidenced by the largest dot diameter in 2022 in Fig. 2), its proportion of international collaborations declined to 18%, reaching its nadir in 2025, as indicated by the green line in Figure 3C.

## 4. Discussion and conclusion

Through a comprehensive, multidimensional bibliometric analysis encompassing keyword distribution, topic evolution, academic influence, and cooperative networks, the critical issue of the disconnect between “pain mechanism” and “clinical translation” in dysmenorrhea research has been distinctly identified. The analysis of keyword and thematic evolution reveals that terms associated with pain mechanisms, such as “dysmenorrhea” and “primary dysmenorrhea,” are central to the research focus, exhibiting prominence with a font size 3 to 4 times larger than other terms. These terms emphasize foundational investigations, including prostaglandin regulation and neuro-immune-endocrine networks. In contrast, clinically translational themes like “quality of life” and “pain management” have been historically marginalized, only appearing in statistical analyses post-2020, with a frequency <50% of that of the core terms. This disparity is further corroborated by the topological structure of the thematic co-occurrence network.

The investigation into pain mechanisms constitutes a central cluster within the “dysmenorrhea → pain → endometriosis” framework, exhibiting a centrality of 0.71. In contrast, the theme of clinical transformation requires traversing 3 levels of transitions to establish associations, with a minimum edge weight of 1.2, and its evolutionary trajectory remains isolated, as indicated by a Jaccard association strength of <0.1. An analysis of academic influence and collaborative networks has identified the structural root cause of this disconnection: the intensity of mutual citation between basic and clinical research clusters is merely 0.03, and cross-cluster connections account for only 12%, significantly lower than the intra-cluster average of 68%. China leads in output, with 38.7% of corresponding authors, yet this output predominantly circulates within domestic journals, creating a closed cycle. The research on pain mechanisms is notably deficient in clinical validation stages, resulting in a “high output − low impact” paradox, with the average annual citation rate projected to decline to zero by 2025. Countries such as Iran have attained a peak citation rate of 2.4 by concentrating on mechanical-translational research, including areas like nonsteroidal anti-inflammatory drug resistance and adolescent pain intervention. However, the global cooperation network exhibits structural deficiencies. In China, the domestic cooperation density (0.48) is 8 times greater than that of international cooperation. Furthermore, collaboration across institutions and borders remains insufficient, hindering the effective integration of knowledge on pain mechanisms with clinical requirements.

To address the disconnect between “pain mechanisms and clinical translation,” a 3-tiered intervention strategy should be adopted. In the short term (1–3 years), efforts should concentrate on disrupting the existing closed cycle. This can be achieved by promoting the submission of research on pain mechanisms to clinical translational journals, with the aim of having international translational journals comprise over 40% of these submissions. Additionally, priority should be given to funding research focused on “mechanism-oriented clinical interventions,” such as personalized treatments via the prostaglandin pathway and studies verifying the neuroimmune mechanisms underlying the efficacy of acupuncture.

In the medium term (3–5 years), efforts should focus on reconstructing the knowledge transmission channel and mandating the integration of basic research projects within the clinical transformation module. This includes the establishment of a joint laboratory dedicated to “pain mechanism − clinical transformation” and the promotion of cross-cluster collaboration. The objective is to enhance the correlation between the mechanism and transformation topics to exceed 0.5. Additionally, a reform of the evaluation system is necessary, ensuring that translational indicators, such as clinical trial outcomes and improvements in patient health, are given equal importance alongside the outputs of basic research. In the long term (5–10 years), the development of a global transformation collaboration network is envisaged. This will leverage the South-South cooperation model, combining China’s expertise in mechanism research with Iran’s experience in transformation. The collaboration will extend to underserved regions such as Africa and the Middle East, with the aim of increasing the node degree of the Iranian network to over 10. Concurrently, a global dysmenorrhea research data platform will be established to consolidate mechanism data, clinical trial results, and real-world outcomes. Expedite the translation of pain mechanism research into clinical guidelines and treatment protocols to effectively address the issue of disconnection.

## Acknowledgments

The authors would like to express their gratitude to the PubMed database for providing the relevant literature data. They also acknowledge the developers of the R language and the bibliometrix software package for offering powerful tools for bibliometric analysis. Additionally, thanks are extended to all researchers whose work is cited in this study, as their contributions laid the foundation for this bibliometric analysis.

## Author contributions

**Conceptualization:** Yuying Zhang, Jinwen Sima, Xinyao Li, Shijia Lu, Yifei Chen, Minghua Dong.

**Data curation:** Yuying Zhang, Jinwen Sima, Xinyao Li, Shijia Lu, Yifei Chen, Minghua Dong.

**Formal analysis:** Yuying Zhang, Jinwen Sima, Xinyao Li, Shijia Lu, Yifei Chen, Minghua Dong.

**Funding acquisition:** Yuying Zhang.

**Visualization:** Yuying Zhang, Jinwen Sima.

**Writing** – **original draft:** Yuying Zhang, Jinwen Sima, Xinyao Li, Shijia Lu, Yifei Chen, Minghua Dong.

**Writing** – **review & editing:** Yuying Zhang, Jinwen Sima, Xinyao Li, Minghua Dong.
